# Social Supportive Day Services: A Promising, Yet Underutilized Respite Option for Dementia Caregivers

**DOI:** 10.1093/geroni/igaf122.2278

**Published:** 2025-12-31

**Authors:** Adriana Hernandez, Setarreh Massihzadegan, Caitlin Coyle

**Affiliations:** University of Massachusetts Boston, Boston, Massachusetts, United States; University of Massachusetts Boston, Boston, Massachusetts, United States; University of Massachusetts Boston, Boston, Massachusetts, United States

## Abstract

Social supportive day programs (SSDP) offer socialization and activities in a group setting for individuals who require a more structured setting, but need minimal assistance with activities of daily living. SSDPs primarily serve older adults with Alzheimer’s disease and related dementias (ADRD), as well as those with physical and functional limitations. Along with positive physical and psychological outcomes for participants, SSDPs provide a form of respite for caregivers. Although promising, utilization rates of SSDPs are varied as are the models of delivery. SSDP may be an essential respite option for dementia caregivers if adequate attention, funding, and resources is given to these programs. In this paper we detail recent efforts by researchers to assess demand for SSDP services and identify barriers and facilitators to SSDP participation. Taking a mixed-methods approach, data from both a survey of residents age 50 and older (N = 1,076) and in-depth interviews with current and past participant families, nonparticipant families, and program staff. Results of this analysis indicate that nearly half of caregivers are providing fewer than 10 hours of care per week and still working full-time. As well, lack of awareness about the target population for the program and financial barriers were identified. Implications of these findings for the models of delivery of supportive day programs will be presented. These findings highlight SSDP as an underdeveloped form of respite and a growing demand for more respite options for dementia caregivers, particularly in smaller towns that provide access to SSDP.

